# *In Vitro* Antileukemic Activity of *Xanthosoma sagittifolium* (Taioba) Leaf Extract

**DOI:** 10.1155/2015/384267

**Published:** 2015-06-09

**Authors:** Marina L. C. Caxito, Rachell R. Correia, Anne Caroline C. Gomes, Graça Justo, Marsen G. P. Coelho, Cássia M. Sakuragui, Ricardo M. Kuster, Katia C. C. Sabino

**Affiliations:** ^1^Departamento de Bioquímica, IBRAG, Centro Biomédico, Instituto de Biologia Roberto Alcantara Gomes, Universidade do Estado do Rio de Janeiro (UERJ), Avenida Professor Manoel de Abreu 44, PAPC, 4° Andar, 20550-170 Rio de Janeiro, RJ, Brazil; ^2^Núcleo de Pesquisas de Produtos Naturais, Universidade Federal do Rio de Janeiro (UFRJ), Rio de Janeiro, RJ, Brazil; ^3^Instituto de Biologia, Universidade Federal do Rio de Janeiro (UFRJ), Rio de Janeiro, RJ, Brazil

## Abstract

*Xanthosoma sagittifolium* Schott is a herb of the Araceae family, popularly known as taioba, which is consumed as food in some regions of Brazil, Africa, and Asia. This species has already been evaluated for the antifungal activities. However, based on its potential antitumor activity, the present study further aimed to examine the antitumor, as well as chelation, activity of *X. sagittifolium* leaf extract. Results showed that hydroethanolic extract of *X. sagittifolium* leaves (HEXs-L) exhibits cytotoxic effects against the immortalized line of human T-lymphocytic (Jurkat) and myelogenous (K562) leukemia cells, but not nontumor RAW 264.7 macrophages or NIH/3T3 fibroblasts. HEXs-L inhibited 50.3% of Jurkat cell proliferation, reducing by 20% cells in G2/M phase, but increasing cells in sub-G1 phase, thereby inducing apoptosis by 54%. In addition, HEXs-L inhibited NO production by 59%, as determined by Griess reaction, and chelated 93.8% of free Fe(II), as demonstrated by ferrozine assay. Phytochemical studies were carried out by ESI-MS, identifying apigenin di-C-glycosides as major compounds. Overall, this work revealed that leaf extract of *Xanthosoma sagittifolium* presented chelating activity and *in vitro* antitumor activity, arresting cell cycle and inducing apoptosis of leukemia cells, thus providing evidence that taioba leaves may have practical application in cancer therapy.

## 1. Introduction

Cancer is characterized by uncontrolled growth, invasion, and, sometimes, metastasis [1]. Its incidence grows annually worldwide, and, in Brazil, it is the second leading cause of death. According to the Brazilian National Cancer Institute (NCI), leukemia is a hematologic cancer characterized by abnormal and uncontrolled proliferation of white blood cells, grouped as lymphoid or myeloid cells, occurring in both children and adults. According to the National Cancer Institute [2], 9,370 new cases of leukemia would occur in Brazil, including 5,050 men and 4,320 women, with the potential to cause such pathophysiological changes as anemia, neutropenia, thrombocytopenia, fever, bleeding, osteoarticular pain, fatigue, and dyspnea [2–4].

Epidemiological evidence has pointed to a connection between chronic inflammation and predisposition for cancer development [5], suggesting that the inflammatory microenvironment can increase mutation rates and accompanying cell proliferation [6]. Such activated inflammatory cells serve as a source of high levels of reactive oxygen species (ROS), including superoxide anion, hydroxyl radicals, and hydroperoxide, as well as nitrogen reactive intermediates (NRS), such as nitric oxide (NO) or peroxynitrite. Together, ROS and NRS are capable of inducing genomic instability, cell damage, and/or death [7]. In addition, nitric oxide is an important mediator of chronic inflammation and regulates cell proliferation, survival, migration, angiogenesis, DNA repair, and tumorigenesis [8]. Accordingly, minimizing ROS and NRS production could protect normal cells from oxidative damage and tumorigenesis [9].

On the other hand, iron is essential for cell growth and proliferation, and perturbation in cellular iron uptake can arrest cell viability, both* in vitro* and* in vivo* [10–12]. Many studies have reported on the potential utility of iron deprivation for the treatment of multiple neoplasias [12–14]. Furthermore, bivalent transition metal ions play an important role as catalysts of oxidative processes, leading to the formation of hydroxyl radicals via Fenton reaction [15].

The identification of new compounds with potential anticancer activity derived from herbaceous foods or medicinal plant extracts has been widely explored in recent years [16], especially those that can serve as immunomodulators or antitumor and antioxidant agents.* X. sagittifolium* Schott, commonly known as taioba, tannia, or cocoyam, is an herbaceous plant of the Araceae family widely grown in many parts of Africa, America, and Asia [17]. The leaves of this species are an excellent source of calcium, phosphorus, iron, and some vitamins [18], and they are consumed as food in South America, especially in Brazil [19]. The rhizome is an important source of starch used as part of the diet in the Amazon region [18]. Despite its nutritional value,* X. sagittifolium* Schott is a poorly studied medicinal plant, being primarily used for treatment of bone diseases based on the results of free calcium (Ca^2+^) analyses [20]. In addition, antifungal activity has been demonstrated for the aqueous extract of* X. sagittifolium* [21].

Based on the antifungal activity of* X. sagittifolium* that antifungal extracts can also present antineoplastic activity [22–24] and considering that antitumor action has never been reported in this species this study aimed to determine the antileukemic potential of* X. sagittifolium* leaf extract, as well as its iron-chelating activity and inhibition of nitric oxide production.

## 2. Materials and Methods

### 2.1. Plant Material and Extract Preparation

Material of* X. sagittifolium* was collected at Nova Friburgo, Rio de Janeiro, Brazil, and identified by Dr. Cassia Sakuragui, Botanic Department of Rio de Janeiro Federal University. An exsiccate is deposited in the Herbarium of the Jardim Botânico do Rio de Janeiro, RJ, Brazil (RB 432051). Air-dried and powdered plant material was macerated in ethanol 70%. The rhizomes (HEXs-R) and leaves (HEXs-L) hydroethanolic extracts were obtained after filtration and solvent evaporation at 60°C (rotary evaporator, Pemen, with vacuum pump Cole Parmer, Instrument Company, Model 7049-50, and a bath, Fisatom, Brazil) and lyophilization (Labconco, Brazil). The extracts were stored at −20°C until use. For cell culture assays, the extracts were dissolved (50 mg/mL) in dimethyl sulfoxide (DMSO) and stored at −20°C. Each extract was diluted in RPMI-1640 medium supplemented with 10% fetal calf serum (RPMI supplemented medium) before experiments.

### 2.2. Cell Culture

Jurkat cells (T-lymphocytic leukemia cell line) were kindly donated by the Basic Research Center of Cancer National Institute of Rio de Janeiro, Rio de Janeiro, Brazil, while K562 cells (myelogenous leukemia cell line), NIH-3T3 (nontumor fibroblast cell line), and RAW-264.7 (macrophage cell line) cells were purchased from Rio de Janeiro Cell Bank, Brazil. The leukemia and NIH-3T3 cells were cultured in RPMI-1640 medium supplemented with 10% fetal calf serum (FCS). RAW 264.7 cells were maintained in DMEM supplemented with 10% FCS and 15 mM HEPES. The cells were expanded two-three times a week. Cultures were incubated at 37°C in humidified atmosphere with 5% CO_2_. Maximal DMSO concentration used in the experimental conditions was 0.05%. Cultures containing medium with DMSO did not present cytotoxic effects.

### 2.3. MTT Cytotoxicity Assay

Leukemia cells (1 × 10^5^/mL) were cultured for 46 h (96-well plate) with different extract concentrations and the cytotoxicity was determined by the MTT [3-(4,5-dimethylthiazol-2-yl)-2,5-diphenyltetrazolium bromide] method [25]. RAW 264.7 (5 × 10^5^ cells/mL) and NIH-3T3 cells (1 × 10^5^ cells/mL) were seeded at 96-well plate and previously incubated 24 h for adhesion. Subsequently, NIH-3T3 cells were incubated for more 46 h with HEXs-L (10–30–50–70–100 *μ*g/mL), and RAW 264.7 cells were incubated for more 22 h with HEXs-L (10–30–50–70–100 *μ*g/mL), both in the presence of* E. coli* lipopolysaccharide (LPS) at 1 *µ*g/mL. After cell incubation with plant samples, each well received 10 *µ*L of MTT (5 mg/mL stock solution) and was incubated for 2 h at 37°C and 5% CO_2_. The formazan crystal was dissolved adding 100 *μ*L/well of 10% sodium dodecyl sulfate solution with HCl 0.01 N. The absorbance was measured at 570 nm after 24 h incubation at 37°C (microplate reader *μ*Quant, Bio-Tek Instruments, Inc.) and viability to each cell line was determined.

### 2.4. Cell Cycle Analysis

Jurkat cells (1 × 10^5^/mL) were cultured in the absence or presence of HEXs-L 50 *µ*g/mL or MTX 2 *µ*g/mL for 48 h. After incubation, the viable cell number was determined (by Trypan blue dye exclusion) and the cell cycle evaluated [26]. After centrifugation, cells (1 × 10^6^) of each experimental condition were suspended in 43 mM citrate buffer (500 *µ*L), containing propidium iodide (PI) 50 *μ*g/mL and Triton X-100 0.3%, homogenized, and remained at room temperature for 15 min in the dark. RNase (100 *μ*g/mL in 43 mM citrate buffer) was added (500 *µ*L/experimental condition) and incubation proceeded for 15 min at room temperature. PI fluorescence (FL-3 channel) was analyzed by flow cytometry on a Gallios Cytometer (Beckman Coulter). Results were analyzed using Summit 4.3v software.

### 2.5. Annexin V Apoptosis Assay

Jurkat cells (1 × 10^5^/mL) were cultured in supplemented RPMI 1640 in the absence or presence of HEXs-L 50 *µ*g/mL or MTX 2 *µ*g/mL for 48 h at 37°C and 5% CO_2_ and humidified atmosphere. Apoptotic cells were labelled using the Annexin V-FITC Apoptosis Detection Kit (BioLegend). Cells (1 × 10^6^) were washed with phosphate buffered saline, pH 7.4 (PBS), and suspended in 500 *µ*L of “binding buffer.” Subsequently, 100 *µ*L of this solution (1 × 10^5^ cells) was transferred into another tube and Annexin V-FITC and PI (100 *µ*g/mL stock solution) were added. The reaction was maintained for 15 min in ice. After this time, “binding buffer” was added (400 *µ*L/tube), and the cells were analyzed by flow cytometry. The fluorescence was detected (5 × 10^4^ events) on a Gallios Cytometer (Beckman Coulter). Data were analyzed using the Summit 4.3v software.

### 2.6. Iron (II)-Chelating Activity

This assay was measured by the decrease of iron(II)-ferrozine complex [27]. The EEXs-L (100 *µ*L) was added to an aqueous solution (140 *µ*L) of 2.0 mM FeCl_2_·4H_2_O. After 10 min, the reaction was initiated by addition of 10 *µ*L of 5.0 mM ferrozine solution. The absorbance at 562 nm was recorded after 10 min of reaction. Quercetin was used as a positive control of chelating activity. The negative control tube contained all the reagents, except the HEXs-L or quercetin, and presented the highest absorbance at 562 nm. The blank tube contained reagents and HEXs-L or quercetin (except ferrozine). Chelating activity (%) was determined based on the formula

(1) Chelating  activity%=Abs  control−Abs  sample−Abs  blankAbs  control×100.

### 2.7. Nitric Oxide Production

Nitric oxide was indirectly determined by measuring nitrite content in the RAW 264.7 macrophage culture supernatant using Griess reagent [28]. Cells (5 × 10^5^/mL) were previously cultured in a 96-well plate for 24 h for adhesion. They were then treated with LPS (1 *μ*g/mL) and different HEXs-L concentrations (25–50–100 *μ*g/mL) for more 24 h. The supernatant cell culture (50 *μ*L) was taken from each well and incubated with 25 *μ*L of sulfanilic acid for 10 min in the dark at room temperature. Then, 25 *μ*L of N-(1-naphthyl)-ethylenediamine was added and the plate was incubated again for 10 min at room temperature in the dark. Finally, the absorbance was read at 550 nm (microplate reader *μ*Quant, Bio-Tek Instruments, Inc.).

### 2.8. Phytochemical Analysis

The chemical characterization of the HEXs-L was achieved by electrospray ionization mass spectrometry (ESI-MS). LCQ equipment Fleet (Thermo Scientific) was used, equipped with electron-spray source operating in negative mode with the analyzer ion trap type. The samples were diluted in 200 *µ*L methanol for 10 mg/mL concentration and injected by direct insertion. The source temperature was set at 180°C, the drying gas (nitrogen) flow rate was 4.0 L/min, and the nebulizer gas (nitrogen) pressure was 0.4 bar. In negative mode, the capillary voltage was 3.8 kV, the capillary exit voltage was −150 V, and the skimmers 1 and 2 voltages were 50 V and 15 V, respectively. Mass calibration was achieved by infusing ammonium formate in an isopropanol-water mixture (1 : 1, v/v) as an external standard. The Xcalibur software was used to obtain spectra in the range of *m*/*z* 100–1500. The detector was a mass spectrometer ion trap type equipped with an interface ESI and analyses were performed using a micrOTOF II mass spectrometer (Bruker Daltonics, Inc., Boston, MA, USA).

### 2.9. Statistical Analysis

The variance analysis was assessed by one-way ANOVA. Differences were considered statistically significant when *p* < 0.05, using Tukey's posttest for differences between three or more experimental groups and Student's *t*-test between two groups. The statistical comparisons were performed using the GraphPad Prism software, version 5.0 (GraphPad Software Inc., San Diego, USA).

## 3. Results

### 3.1. The* In Vitro* Cytotoxicity of* X. sagittifolium* Extracts

**Figure 1:**
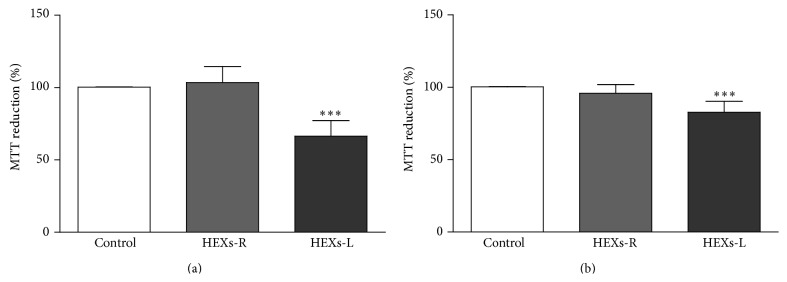
Cytotoxic effects of HEXs on leukemic cells. (a) Jurkat cells. (b) K562 cells. Cells (1 × 10^5^/mL) were incubated in the absence (control) or presence of rhizome (HEXs-R) or leaf extracts (HEXs-L) at 50 *µ*g/mL for 48 h. Cytotoxicity was determined by the MTT assay. The results express mean percentage of control ± S.D. of three experiments with triplicates. ^*∗∗∗*^ *p* < 0.001, related to control, by one-way ANOVA followed by Tukey's posttest.

**Figure 2:**
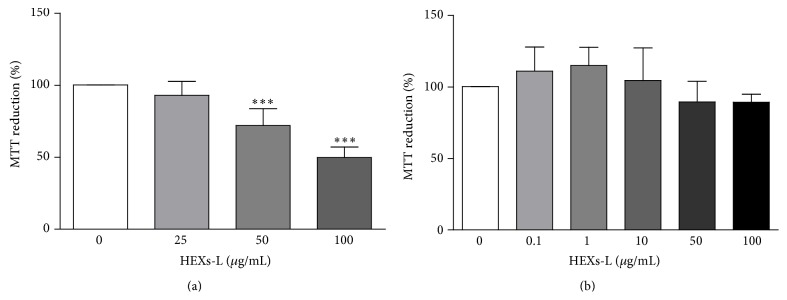
Cytotoxic effects of HEXs-L on leukemia and normal cells. (a) Jurkat cells. (b) Nontumor NIH fibroblasts. Cells were incubated in the absence (control) or presence of HEXs-L for 48 h. Cytotoxicity was determined by the MTT assay. The results express mean percentage of control ± S.D. of three experiments with triplicates. ^*∗∗∗*^ *p* < 0.001, related to control, by one-way ANOVA followed by Tukey's posttest.

To investigate the effect of* X. sagittifolium* extracts on leukemia cells, the cytotoxicity of both leaf (HEXs-L) and rhizome (HEXs-R) extracts (50 *µ*g/mL) was evaluated on lymphocytic (Jurkat) and myelocytic (K562) leukemia cells. The results, as shown in Figure 1, indicated that treatment for 48 h with HEXs-L partially inhibited mitochondrial reduction activity (MRA) of both leukemia cell lines, with Jurkat cells (Figure 1(a)) presenting higher inhibition index (33.6 ± 10.8%) than K562 cells (17.3 ± 7.5%) (Figure 1(b)). HEXs-R was not cytotoxic against leukemia cells. The effects of other HEXs-L concentrations (25–100 *μ*g/mL) were evaluated as well, presenting IC_50_ of 95.9 *μ*g/mL (Figure 2(a)). HEXs-L showed no cytotoxicity against NIH/3T3 fibroblasts (Figure 2(b)).

### 3.2. Effects of HEXs-L on Jurkat Cell Proliferation and Apoptosis

**Figure 3:**
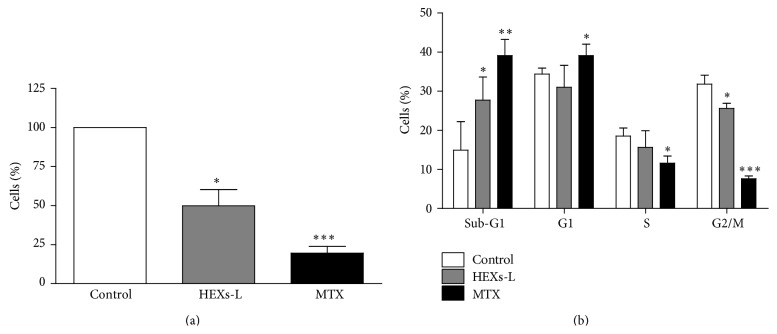
Effects of HEXs on Jurkat cell proliferation. (a) Viable cell number. (b) Cell cycle analysis. Cells (1 × 10^5^/mL) were treated with HEXs-L (50 *µ*g/mL) or MTX (2 *µ*g/mL) or culture medium (control) for 48 h and then counted by contrast phase microscopy and processed for cell cycle analysis by flow cytometry, as described in Materials and Methods section. The results express mean ± S.D. of five independent experiments. ^*∗*^ *p* < 0.05, ^*∗∗*^ *p* < 0.01, and ^*∗∗∗*^ *p* < 0.001, related to control, by one-way ANOVA followed by Tukey's posttest.

**Figure 4:**
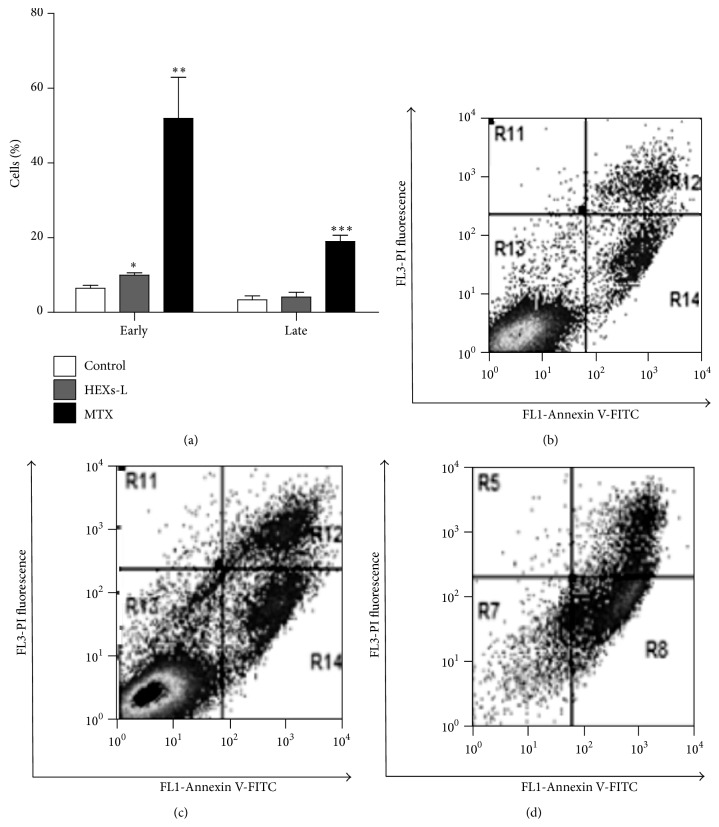
Effects of HEXs-L on Jurkat cell apoptosis by annexin-V-FITC labeling assay. (a) Early and late apoptotic cells (mean of three independent experiments). Cytograms of a representative experiment are shown in (b) control, (c) HEXs-L, and (d) MTX treated cultures. Cells (1 × 10^5^/mL) were treated with HEXs-L (50 *µ*g/mL) or MTX (2 *µ*g/mL) or culture medium (control) for 48 h and then processed for flow cytometric analysis of apoptosis as described in Materials and Methods section. The results in (d) express mean ± S.D. ^*∗*^ *p* < 0.05, ^*∗∗*^ *p* < 0.01, and ^*∗∗∗*^ *p* < 0.001, related to control, by one-way ANOVA followed by Tukey's posttest.

Treatment with HEXs-L for 48 h inhibited Jurkat cell proliferation, reducing significantly (50.7%) the viable cell number, compared to control (Figure 3(a)), as observed with methotrexate- (MTX) (80.5%) treated cultures. This HEXs-L effect was accompanied by an increase (85.9%) of cells in the sub-G1 phase and a reduction (20.0%) of cells in the G2/M phase (Figure 3(b)). These effects in the cell cycle were also observed with the MTX treatment, although at higher level than the plant extract (Figure 4(b)). In addition, MTX increased cells in the G1 phase (13%) and reduced them in the S phase (38%), compared to HEXs-L, 85.9% and 20%, respectively, as noted above.

Investigating the effects of HEXs-L (50 *µ*g/mL) on cell death, the percentage of early apoptotic cells was increased (54.2%), compared to control culture, while MTX induced a higher level of early (7-fold) and late (4.6-fold) apoptosis, compared to control or HEXs-L treatment (Figure 4(a)). Cytograms of a representative apoptosis experiment are also shown in Figures 4(b)–4(d).

### 3.3. Iron Chelation Activity and NO Production

**Figure 5:**
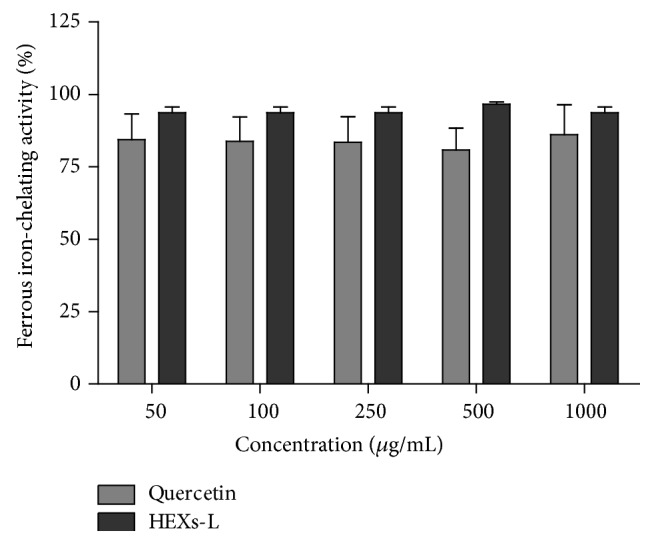
Effects of HEXs-L on iron(II)-chelating activity by the ferrozine assay. Quercetin was used as a chelating activity positive control. The results express mean ± S.D. of three experiments with triplicates. There were no significant differences between HEXs-L and quercetin, by Student's *t*-test.

**Figure 6:**
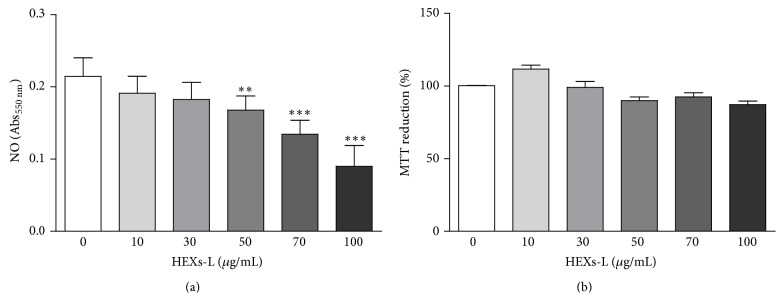
Effects of HEXs-L on RAW 264.7 macrophages. (a) NO production. (b) Cytotoxicity. Cells (5 × 10^5^/mL) were treated or not with HEXs-L for 24 h and NO was then indirectly estimated in culture supernatant by nitrite determination (Griess reaction) and cell cytotoxicity was evaluated by MTT assay. The results express mean ± S.D. of three experiments with triplicates. ^*∗*^ *p* < 0.05 and ^*∗∗∗*^ *p* < 0.001, related to control, by one-way ANOVA followed by Tukey's posttest.

The inflammation microenvironment, rich in reactive oxygen (ROS) and NO/peroxynitrite, and cancer promotion have been linked. The antioxidant property of HEXs-L was then investigated by ferrous iron-chelating ability, since Fe^2+^ can lead to hydroxyl radical (OH^∙^) production via Fenton reaction. The results (Figure 5) indicated a high ferrous iron-chelating ability for HEXs-L that did not differ significantly from that of quercetin, the positive control of ferrous iron-chelating activity used in this assay. HEXs-L also reduced NO production by RAW 264.7 macrophages in a concentration-dependent manner (Figure 6(a)), showing IC_50_ of 86.3 *µ*g/mL, without cytotoxic effects (Figure 6(b)).

### 3.4. Phytochemical Analysis of the HEXs-L

**Figure 7:**
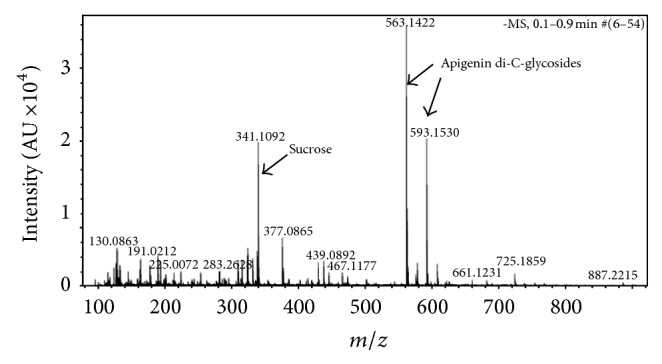
The electron-spray ionization (ESI) mass spectrum of 70% hydroalcoholic extract of* Xanthosoma sagittifolium* leaves. MS data were acquired in the negative ionization mode. The mass detector was an ion trap spectrometer equipped with an ESI interface. All data were analyzed using Bruker Daltonics ESI Compass Data Analysis version 4.0 SP1 (Bruker Daltonics Inc., MA, USA).

The chemical analysis of HEXs-L showed two substances with molecular weight of 563.1422 and 593.1530 and molecular formulae of C_26_H_27_O_14_ and C_27_H_29_O_15_, respectively, as major compounds, proposed as apigenin di-C-glycosides, as well as sucrose, pyroglutamic acid (5-oxoproline), and some fatty acids (palmitic, stearic, and linolenic acids) by ESI-MS (Figure 7).

## 4. Discussion

The present study investigated the potential effects of* X. sagittifolium* Schott extract on the* in vitro* growth of leukemia cells, NO production, and iron-chelating activity. Mitochondria play a crucial role in cell survival and function by generating most of the cell's supply of adenosine triphosphate (ATP) [29]. The inhibition of mitochondrial reduction activity by the effect of HEXs-L on leukemia cells, as observed in this work, may be related to cytotoxic substances in the leaf, but not the rhizome (HEXs-R), since even higher concentrations of HEXs-R were not toxic. Leaves of plants of the* Xanthosoma* genus have previously been recognized as containing cytotoxic compounds, showing bactericidal activity, for example,* X. robustum* extract [30], and antifungal property, for example,* X. sagittifolium* extract [21]. In addition, aerial parts of plants have been used as popular medicines to treat a broad range of diseases, including cancer [31]. The toxicity of HEXs-L to leukemia cells does not result from dimethyl sulfoxide (DMSO), since control cultures with this solvent did not alter the responses (data not shown).

The lower sensitivity of myeloid K562 leukemia cells, compared to lymphocytic Jurkat cells, to the cytotoxic effects of HEXs-L, can be attributed to the lower expression of ABCG2 receptor in Jurkat cells, an ABC membrane transporter that induces drug resistance [32]. It is important to note that the treatment of nontumor NIH/3T3 fibroblasts with HEXs-L did not reduce their viability, which is an important finding since many antineoplastic drugs are extremely cytotoxic to nontumor cells [33]. The extract of other Araceae plants, such as* Rhaphidophora korthalsii* Scott [34] and* Typhonium blumei* Nicolson & Sivad [35], did not present cytotoxic effects to normal cells or affect the sensitivity of normal cells to a lesser extent than tumor cells, respectively. Or cytotoxicity to normal cells is less significant.

Since HEXs-L was demonstrated to reduce mitochondrial function in tumor cells, we evaluated if the extract could also inhibit Jurkat cell proliferation or only its activation. It was found that HEXs-L significantly reduced the number of Jurkat cells in the culture. This inhibitory effect on leukemia cell proliferation was corroborated by a decrease in the percentage of cells in the G2/M phase.

Iron is essential for neoplastic cell proliferation; it participates in DNA synthesis and macromolecule biosynthesis; accordingly neoplastic cells have a high iron requirement [36]. In the absence of iron, cells are unable to progress in the cell cycle. Recently, the role of iron chelation as antitumor agents has been investigated by targeting ribonucleotide reductase, the enzyme responsible for deoxyribonucleotide synthesis, which is the iron dependent enzyme involved in the rate limiting phase of DNA synthesis [37–39]. Therefore, we also studied the iron-chelating property of HEXs-L, which showed a high level of this activity. Meanwhile, other studies have shown that treatment with HEXs-L may result in a lower concentration of cellular deoxyribonucleotides in tumor cells, impairing their ability to complete the S phase of cell cycle. Consequently, cell growth and preparation for cell division would be curtailed in G2, leading to unsuccessful mitosis and cytokinesis in M phase, as described for the desferrioxamine, an iron-chelating agent that binds free iron in a stable complex, which is currently used to treat iron-overload disease and tumor cell growth [40–42]. Since several reports have also revealed the antiproliferative activity of desferrioxamine [43, 44], this hypothesis needs further investigation, which, however, is beyond the scope of the present report.

The increased number of leukemia cells in the sub-G1 phase after treatment with HEXs-L is an indication of DNA fragmentation in these cells. Since apoptosis is frequently associated with cell cycle arrest [45] and since apoptotic cells also show DNA fragmentation, we decided to investigate the effects of HEXs-L on the induction of apoptosis. Apoptosis is also characterized by other biochemical events, such as surface exposure of phosphatidylserine phospholipid (Ptd-L-Ser), mitochondrial membrane permeabilization, and activation of caspases [46]. Therefore, Jurkat cell apoptosis was evaluated by the surface exposure of Ptd-L-Ser. To accomplish this by performing flow cytometry and based on the increased number of Annexin V-positive cells, HEXs-L treatment was confirmed to induce apoptosis in Jurkat cells. This result is important because hyperproliferative conditions, such as autoimmune disease and cancer, are frequently associated with apoptosis impairment [46] while the induction of apoptosis is found as a mechanism of action for many traditional antineoplastic drugs [47].

Although low concentration of NO mediates important physiological processes [48] high levels play crucial role in antimicrobial and antiviral responses [49] and can trigger oxidative stress, chronic inflammation [50], and nitrosylation, in particular S-nitrosylation of mitochondrial caspase-8, caspase-9, and Bcl-2 results in the inhibition of apoptosis of tumor cells [51]. The reduction of the NO level in the culture supernatant of LPS-stimulated macrophages by treatment with HEXs-L, as described for other plants of the Araceae family [52], may represent an important finding since reduction of nitrosylation of caspases would, in turn, lead to the activation of caspases to cleave cellular organisms in the cell and cause apoptosis, thus contributing to the surveillance of tumor cell promotion/progression. Additionally, NO mediates inflammation and epidemiological studies have shown that chronic inflammation predisposes individuals to different types of cancer [53]. Many studies reporting on cancer development have focused on inflammation as a potential mechanism underlying the disease in its early stages [53]. Inhibition of NO production by HEXs-L can also represent a potential mechanism of inhibiting tumorigenesis.

Redox homeostasis is important for cell survival because it regulates the functions of transcription factors, signal transduction pathways, and mediators of cell proliferation and death [54]. High levels of ROS and NRS lead to oxidative stress and deleterious modifications to DNA, lipids, and proteins [55], inducing chronic inflammation and, potentially, cancer. Thus, the presence of free Fe^2+^, for example, can, through Fenton's reaction, lead to hydroxyl radical (OH^∙^) production, contributing to oxidative stress [56]. Furthermore, NO can react with superoxide, which results in the production of peroxynitrite, a more powerful oxidant. In this context, HEXs-L presents the property of chelating free Fe^2+^ which can promote antitumor activity by reducing ribonucleotide reductase activity and DNA synthesis, as well as reducing cell damage by oxidative stress, thus avoiding chronic inflammation and/or cancer development [56].

The use of plants as a source of phytochemical compounds in therapeutic medicine is increasingly being recognized [57]. In addition, flavonoids can affect the cell cycle regulation of cancer cells and apigenin glycoside has been found to be one of them [58]. The phytochemical analysis of HEXs-L by ESI-MS identified apigenin diglycosides as major compounds, corroborating reports [59, 60] that plants of this family are rich in apigenin and other flavonoids. Interestingly, apigenin and apigenin derivatives have previously been reported to inhibit the proliferation of leukemia cells [47, 61] chelating free Fe^2+^, induce leukemia apoptosis [62], and inhibit NO production [63]. Flavonoids are a group of natural compounds found in plants with variable phenolic structures. Their mechanisms of action can include scavenging superoxide, H_2_O_2_, nitric oxide, and hydroxyl radicals [64, 65]. Furthermore, flavonoid antioxidant capacity involves combined effect of radical scavenging activity, interaction with enzyme functions, or chelating elements involved in free radical generation [65]. In summary,* Xanthosoma sagittifolium* contains apigenin glycosides as major compounds and shows important biological activities, including Fe^2+^ chelation, inhibition of NO production, and inhibition of leukemia cell proliferation, mechanisms reported for traditional antitumor agents [66]. However, while antitumor activity may result from the presence of apigenin glycosides in the HEXs-L, it is also likely that other phytochemical compounds in the extract could be associated with this activity, as well as other biological effects noted above.

## 5. Conclusions

For the first time, the present work describes the antitumor activities of hydroethanolic extract of* Xanthosoma sagittifolium* Schott (taioba) leaves, including Fe^2+^ chelation, inhibition of NO production, and inhibition of leukemia cell proliferation. These properties may principally result from the presence of apigenin glycosides in HEXs-L. Therefore, by revealing the chelating activity of* Xanthosoma sagittifolium* leaf extract, as well as its antitumor activity, including cell cycle arrest and the induction of apoptosis, this work has provided evidence that taioba leaves may have practical application in cancer therapy.
